# Differential Effects of Cold Atmospheric Plasma in the Treatment of Malignant Glioma

**DOI:** 10.1371/journal.pone.0126313

**Published:** 2015-06-17

**Authors:** Alan Siu, Olga Volotskova, Xiaoqian Cheng, Siri S. Khalsa, Ka Bian, Ferid Murad, Michael Keidar, Jonathan H. Sherman

**Affiliations:** 1 Department of Neurological Surgery, George Washington University, Washington, DC, United States of America; 2 Department of Mechanical and Aerospace Engineering, George Washington University, Washington, DC, United States of America; 3 George Washington University School of Medicine and Health Sciences, Washington, DC, United States of America; 4 Department of Biochemistry and Molecular Biology, George Washington University, School of Medicine, Washington, DC, United States of America; University Paul Sabatier, FRANCE

## Abstract

**Objective:**

Cold atmospheric plasma (CAP) has recently been shown to selectively target cancer cells with minimal effects on normal cells. We systematically assessed the effects of CAP in the treatment of glioblastoma.

**Methods:**

Three glioma cell lines, normal astrocytes, and endothelial cell lines were treated with CAP. The effects of CAP were then characterized for viability, cytotoxicity/apoptosis, and cell cycle effects. Statistical significance was determined with student's t-test.

**Results:**

CAP treatment decreases viability of glioma cells in a dose dependent manner, with the ID50 between 90-120 seconds for all glioma cell lines. Treatment with CAP for more than 120 seconds resulted in viability less than 35% at 24-hours posttreatment, with a steady decline to less than 20% at 72-hours. In contrast, the effect of CAP on the viability of NHA and HUVEC was minimal, and importantly not significant at 90 to 120 seconds, with up to 85% of the cells remained viable at 72-hours post-treatment. CAP treatment produces both cytotoxic and apoptotic effects with some variability between cell lines. CAP treatment resulted in a G2/M-phase cell cycle pause in all three cell lines.

**Conclusions:**

This preliminary study determined a multi-focal effect of CAP on glioma cells in vitro, which was not observed in the non-tumor cell lines. The decreased viability depended on the treatment duration and cell line, but overall was explained by the induction of cytotoxicity, apoptosis, and G2/M pause. Future studies will aim at further characterization with more complex pre-clinical models.

## Introduction

Cold atmospheric plasma (CAP) is a partially ionized gas that can focally deliver low levels of ultraviolet (UV) radiation and ionized particles to synergistically generate reactive oxygen species (ROS). This technology has demonstrated efficacy in several biomedical applications ranging from anti-bacterial decontamination to promotion of wound healing [1]. The anti-growth effects of CAP on prokaryotic cells are well established, but its effects on eukaryotic cell remain largely uncharacterized. Lower dosages (i.e. duration or power) of helium-CAP can stimulate detachment, migration, and proliferation in fibroblasts [2], endothelial [3,4] and smooth muscle cells *in vitro* [5,6]. Several studies investigating the role of CAP in various cancers have shown promise [7–10]. In addition, we recently showed that application of CAP can selectively induce apoptosis in various cancer cell lines *in vitro* and significantly reduce *in vivo* tumor size of a murine melanoma model [11]. Our group also recently demonstrated a key mechanism of cell cycle interference in malignant cells [12]. In specific, we have shown that CAP treatment induced a robust increase in the G_2_/M population in epidermal cancer cells but not in non-malignant cells *in vitro*.

Glioblastoma is a highly malignant primary central nervous system neoplasm associated with poor survival and is invariably fatal. It is considered one of the most aggressive forms of human cancers, characterized largely by its rapid growth, extensive angiogenesis, and invariable resistance to all current therapies. Consequently, treatments remain largely palliative despite recent advances with the integration of multi-modal therapies. The current standard of care, known as the Stupp protocol, improves median survival up to 14.6 months [13] with five-year survival at less than 10% [14].

Recurrence of glioblastoma is typically local, due in large part to poor treatment distribution, limited therapeutic penetration, and development of drug resistance to chemotherapeutic agents [15,16]. Current radiation protocols are limited by the side-effect profile due to poor specificity. Bevacizumab remains on the only FDA approved treatment at recurrence with median survival ranging 3.9 to 9.2 months [17–19]. Thus it is necessary to develop novel tools that can provide selective targeting of proliferating cells to enhance current therapies. Cold non-thermal atmospheric plasma can offer a highly precise means of delivering chemically specific modifications to an area such as a resection cavity, with high selectivity for actively proliferating cells. The combination of ROS generation from charged particles and UV could also provide a distinctive advantage in inducing a multi-faceted mechanism of selective cellular injury [20–23]. These characteristics could establish CAP as an adjunctive treatment for glioblastoma. In this preliminary study, we thus sought to characterize the *in vitro* effects of CAP on three distinct glioma cell lines in comparison to normal human astrocytes (NHAs).

## Methods

### Cell Lines and Cell Culture

Glioma cell lines (U87, U373, A172) and human umbilical vein endothelial cells (HUVEC) were obtained from the American Type Culture Collection (Manassas, VA). Human normal astrocytes E6/E7 were generously donated by Dr. Andrew Parsa at The University of California San Francisco. All the cell lines were maintained at 37°C, 5% CO2 in Dulbecco’s modified Eagle’s medium (DMEM) supplemented with 10% fetal bovine serum (HyClone, Logan, UT) plus 1% penicillin/streptomycin mixture. At ~70% confluence, the cells were re-plated onto (1) 96-well plates for the MTT assay and ApoTox-Glo™ Triplex assay, or (2) 12-well plates for cell cycle determination through propidium iodide staining followed by flow cytometry. Experiments were undertaken 48 hours post-plating, at ~40% confluence.

### CAP (Cold Atmospheric Plasma) Jet

**Fig 1:**
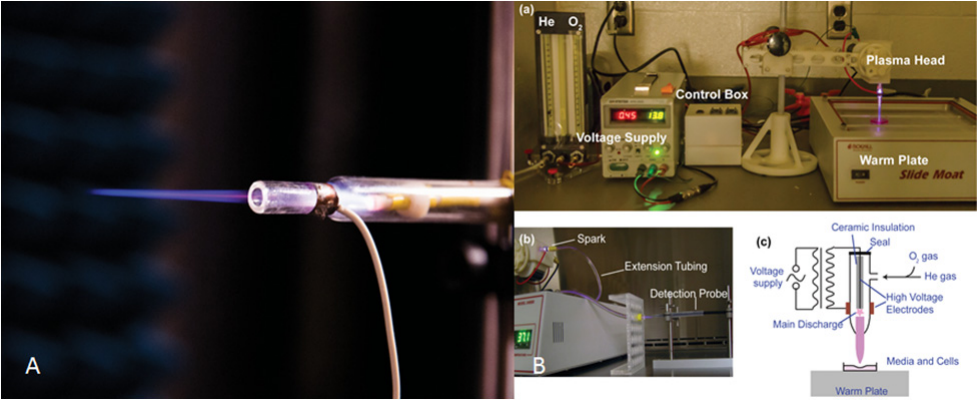
Cold Atmospheric Plasma (CAP) jet setup. (A) Cold atmospheric plasma (CAP) jet, with which can be mounted for treatment directly over culture plate containing various glioma cell lines (U87, U373, A172) for various durations. (B) The parameters of the CAP were an output voltage between 4.5–5 kV, a frequency of 13 kHz and a helium flow rate at 10 L/min. The distance between the jet and the base of the culture plate was maintained at 20 mm, yielding an average jet dose of 0.8 J/sec/cm^2^.

The CAP jet is the dielectric barrier discharge in helium that is described elsewhere [24] (see Fig 1). The parameters of the CAP were an output voltage between 4.5–5 kV, a frequency of 13 kHz and a helium flow rate at 5 L/min. Energy produced in the CAP device is in the order of 100 J in the case of tens of seconds to minutes of treatment. However only a small fraction of the discharge energy is associated with the jet. Indeed it is about 1% of the discharge energy that is deposited in the jet [2,12]. Taking into account the characteristic scale of the jet of about cm, it can be estimated that the dose is about 1J/s per cm^2^.

**Fig 2:**
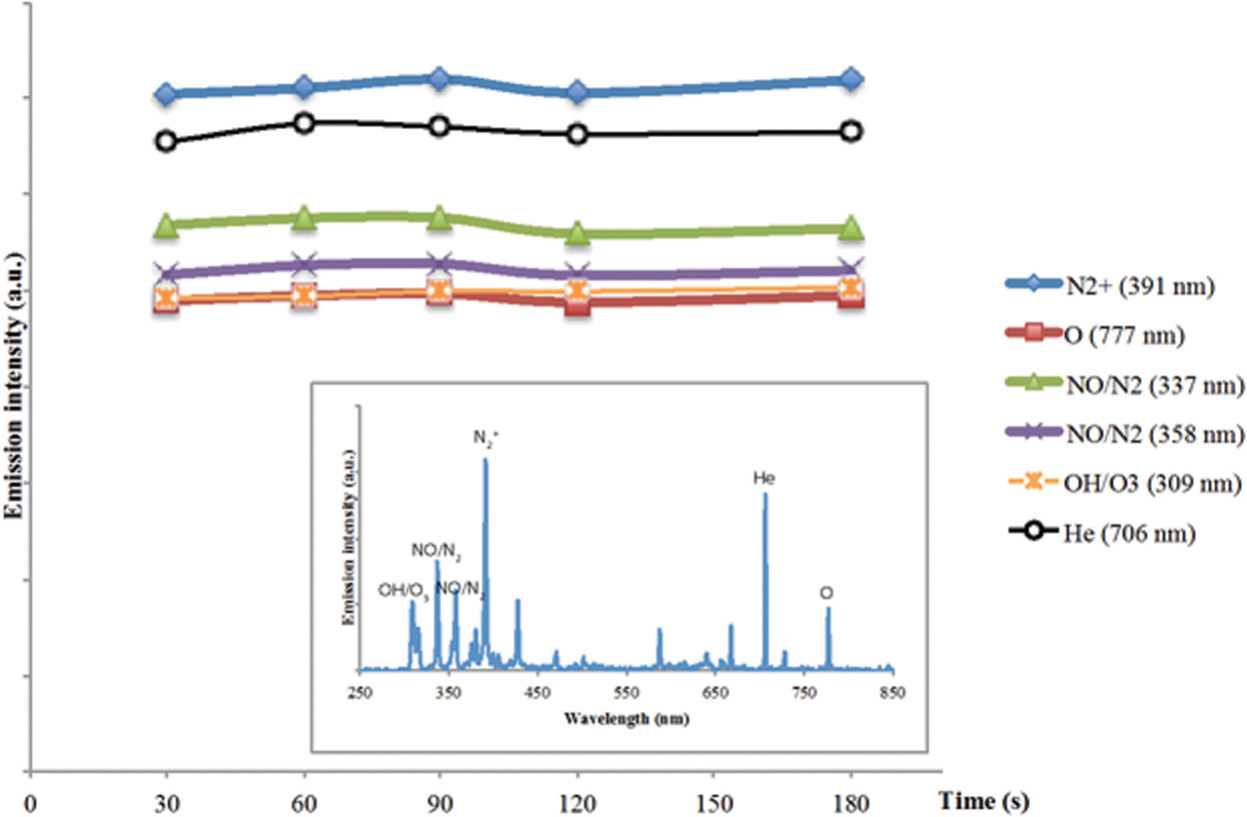
Spectral Characterization of CAP jet. Spectra of reactive species generated by CAP on media (DMEM, 10% FBS, 1% abx), which produces a stable proportion of reactive species over the duration of treatment (30 to 180 seconds).

The ionized nitrogen species and highly reactive oxygen radicals are present in the CAP spectrum, which was stable over time, and consisted largely reactive nitrogen and oxygen species (Fig 2) [25]. We used experimental protocol established in our earlier studies [12] All experiments with cold plasma were conducted at 48 hours post-plating. The cells were all immersed in media during CAP treatment and fresh medium was added to the cells immediately after CAP treatment. All experiments were performed in at least quintuplets and verified a second time.

### MTT Assay

The MTT assay was used to quantify viability of each cell line after treatment with CAP for 60 to 180 seconds. The cells were incubated with 1.7 mM of Thiazolyl Blue Tetrazolium Bromide (Sigma, cat#M2128) for 3 hours at 37°C. This tetrazolium salt is metabolically reduced by the mitochondria of viable cells to yield a blue insoluble Formosan product that is detectable spectrophotometrically. The percentage of surviving treated cells compared to control cells was calculated from the average OD_570_ values obtained in each experiment, and plotted over time. The MTT assay was performed at 24, 48, and 72 hours post-CAP treatment.

Untreated controls were maintained throughout the experiment as a negative control, also measured at 570 nm. The viability of the treated cells were calculated as a percentage this negative control.

### ApoTox-Glo Triplex Assay

The ApoTox-Glo Triplex Assay was purchased from Promega Corp (G6320; Madison, WI). This assay combines three distinct chemistries to assess viability, cytotoxicity, and caspase 3/7 activity within a single well format, as per protocol. Briefly, the simultaneous measurement of two proteases was used as markers of viability and cytotoxicity through glycylphenylalanyl-aminofluorocoumarin (GF-AFC) and bis-alanylalanyl-phenylalanyl-rhodamine 110 (bis-AAF-R110), respectively. GF-AFC is cell permeable and thus enters an intact cell where it is cleaved by a live-cell protease to generate a fluorescent signal proportional to the number of living cells. Conversely, this live-cell protease becomes inactive upon loss of cell membrane integrity and leakage into the surrounding culture medium. Bis-AAF-R110 is used to measure dead-cell protease activity, which is released with loss of cell membrane integrity. As bis-AAF-R110 is not cell-permeable, no signal is generated from intact cells. The simultaneous determinaton of both products was made possible due to different excitation/emission spectra, as measured by the BioTek Synergy H1 Hybrid Microplate Reader. The viability and cytotoxicity were measured at 4, 24, and 48 hours post-CAP treatment.

The second component of the assay utilizes a caspase-Glo™ 3/7 reagent in lysed cells, which produces luminescent signal by luciferase that is measured by a BioTek Synergy H1 Hybrid Microplate Reader. The caspase 3/7 activity was measured at 4, 24, and 48 hours post-CAP treatment.

### Cell Cycle Determination with Flow Cytometry

Each of the glioma cells were plated in 24-well plates. After 48 hours, at ~ 40% confluence, the cells were treated for either 90 or 120 s CAP. After treatment with CAP, the DNA content stained with propidium iodide/RNase staining buffer (BD Pharmingen, cat# 550825) in 0.1% Triton X-100. Cells were trypsinized with 0.25% Trypsin EDTA (Invitrogen, Gibco, cat# 25200), washed with PBS prior to fixation in 70% ethyl alcohol, stored at -20°C for 24 hours and then stained.

DNA content was measured using flow cytometry on a FACSCalibur DxP8 at the GWU Flow Cytometry Core Facility (manufactured by BD Biosciences and upgraded by Cytek Development). The analyzer is equipped with three lasers (providing excitation wavelengths of 488, 637 and 407 nm) and eight detectors for fluorescence. The DNA content was measured at 24 and 48 hours post-CAP treatment.

### Statistical Analysis

Results are expressed as mean ± SEM. A one-way analysis of variance was performed for multiple comparisons, and if there was a significant variation between the treatment groups, the mean values for a treated group were compared with those of the control by Student’s *t* test; *p* values of less than 0.05 were considered statistically significant. The *n* values indicate the numbers used in each experiment. The ID_50_ (inhibitory duration which caused 50% inhibition) was estimated using a linear regression method with Microsoft Excel, 2007.

## Results

### CAP Treatment Decreases Viability of Glioma Cells

**Fig 3:**
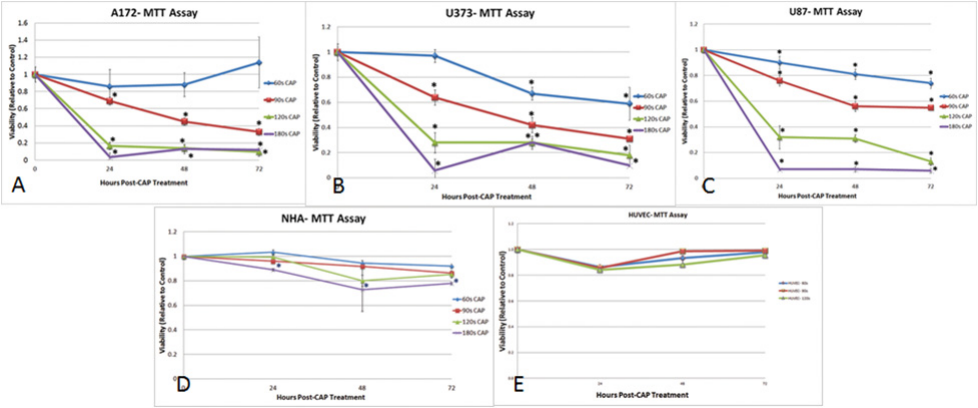
Cell viability under CAP treatment. Results of MTT assay from (A) A172, (B) U373, (C) U87, (D) NHA and (E) HUVEC treated with varying durations of cold atmospheric plasma (CAP). After treatment, the MTT assay was performed at 24, 48, and 72 hours. Viability was calculated relative to control. All three glioma cell lines exhibited a dose-dependent decline in viability with CAP treatments ranging from 60 to 180 seconds. The effects of CAP were sustained up to 72 hours post-treatment. Compared to the other two cancer cell lines, U87 seemed to be more resistant to CAP treatment for 90 seconds. No significant response was seen with NHA, and only transient effects observed on HUVEC cells. * denotes statistical significance compared to control (P <0.05).

The *in vitro* exposure of all three glioma cell lines resulted in a dose-dependent and predictable decline in viability as assessed by the MTT assay (Fig 3). At maximal treatment duration of 180 seconds, viability decreased to less than 15% compared to control by 24 hours post-treatment for all three cell lines, in contrast to 60 seconds treatment which had only modest effects depending on the cell line (Table 1). Treatment with CAP for 120 seconds was the shortest duration that produced a global decline across all three glioma cell lines. CAP treatment for 90 seconds resulted in sustained decline even at 72 hours post-treatment for A172 and U373 in contrast to U87 which seemed to plateau at 72 hours (Fig 3).

**Table 1:** Comparison of cell viability under Cap treatment in three different glioma cell lines.

MTT Assay Post-Treatment (hours)	Duration of CAP Treatment (seconds)	Glioma Cell Line % Viability (SEM)		
		A172, (n = 22)	U373, (n = 22)	U87 (n = 22)
24	60	86 (0.09)	97 (0.04)	90 (0.05)*
	90	69 (0.03)*	64 (0.06)*	76 (0.04)*
	120	17 (0.04)*	28 (0.07)*	32 (0.09)*
	180	4 (0.04)*	6 (0.01)*	7 (0.01)*
48	60	88 (0.2)	67 (0.05)*	81 (0.04)*
	90	45 (0.1)*	42 (0.06)*	56 (0.04)*
	120	14 (0.03)*	28 (0.08)*	31 (0.03)*
	180	13 (0.04)*	28 (0.09)*	7 (0.02)*
72	60	114 (0.14)	59 (0.05)*	74 (0.04)*
	90	33 (0.04)*	31 (0.06)*	55 (0.02)*
	120	9 (0.02)*	18 (0.05)*	13 (0.03)*
	180	12 (0.05)*	10 (0.03)*	6 (0.02)*

Results of MTT assay from three glioma cell lines, A172, U373, and U87 treated with varying durations of cold atmospheric plasma (CAP). After treatment, the MTT assay was performed at 24, 48, and 72 hours. Viability was calculated relative to control.

*Denotes significance to p< 0.05 relative to control.

**Fig 4:**
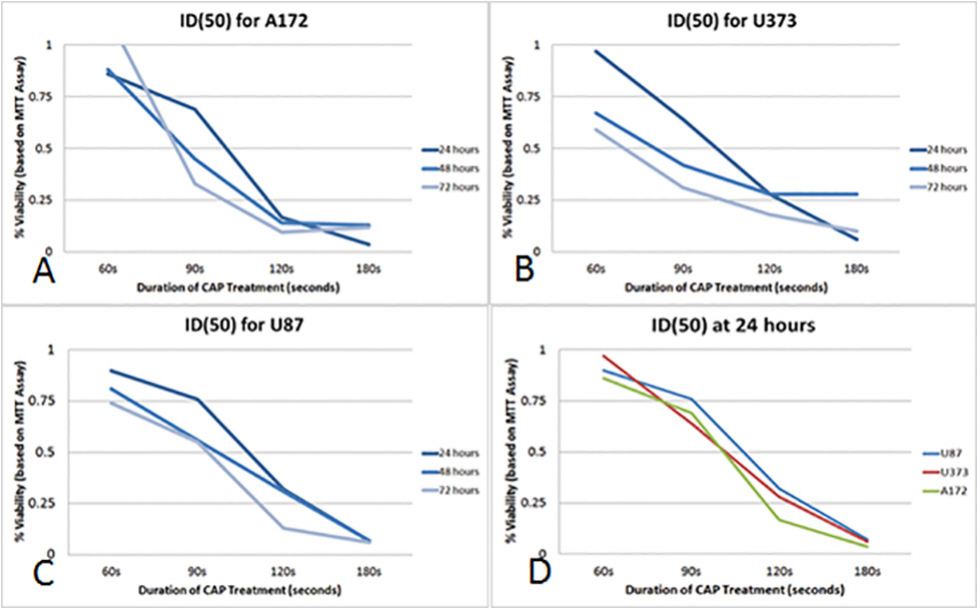
Inhibitory dose of CAP (ID_50_). Duration of CAP treatment necessary to decrease viability by 50%, termed the inhibitory duration (ID_**50**_). The ID_**50**_ for (A) A172, (B) U373, and (C) U87 is between a treatment duration of 90 to 120 seconds with CAP. The ID_**50**_ for A172 and U373 declined at 48 and 72 hours more significantly than in U87, indicating a relative treatment resistance to CAP in U87. (D)The ID_**50**_ for all cell lines are between 90 to 120 seconds at 24 hours.

From this data, the duration of treatment necessary to decrease the viability by 50% (ID_50_) was calculated for each cell line at 24, 48 and 72 hours post-treatment (Fig 4). At 24 hours post-treatment, the ID_50_ was seen at between 90 and 120 seconds for all three cell lines. At 48 and 72 hours post-treatment, the ID_50_ for treatment duration declined for U373 and A172, but remained relatively stable for U87 displaying a higher duration of treatment to achieve a 50% reduction in viability in the U87 cell line. Overall, the ID_50_ was achieved with a treatment duration of between 90 to 120 seconds.

### CAP Treatment Does Not Decrease Viability of NHA and HUVEC Cells

In contrast to the glioma cells, CAP treatment did not significantly decrease the viability on NHA and HUVEC cells as assessed by the MTT assay. At the maximal treatment duration of 180 seconds, the viability of the NHA remained 78% at 72 hours post-treatment. No significant decline in viability was observed for treatment times between 60 to 120 seconds in NHA cells (Fig 3D).

A transient but significant decline to >85% viability was observed at 24 hours with CAP treatments of 60 to 120 seconds, followed by reconstitution to greater than 95% viability by 72 hours (Fig 3E).

### CAP Treatment Results in Both Cytotoxic and Apoptotic Effects in Glioma Cells

**Fig 5:**
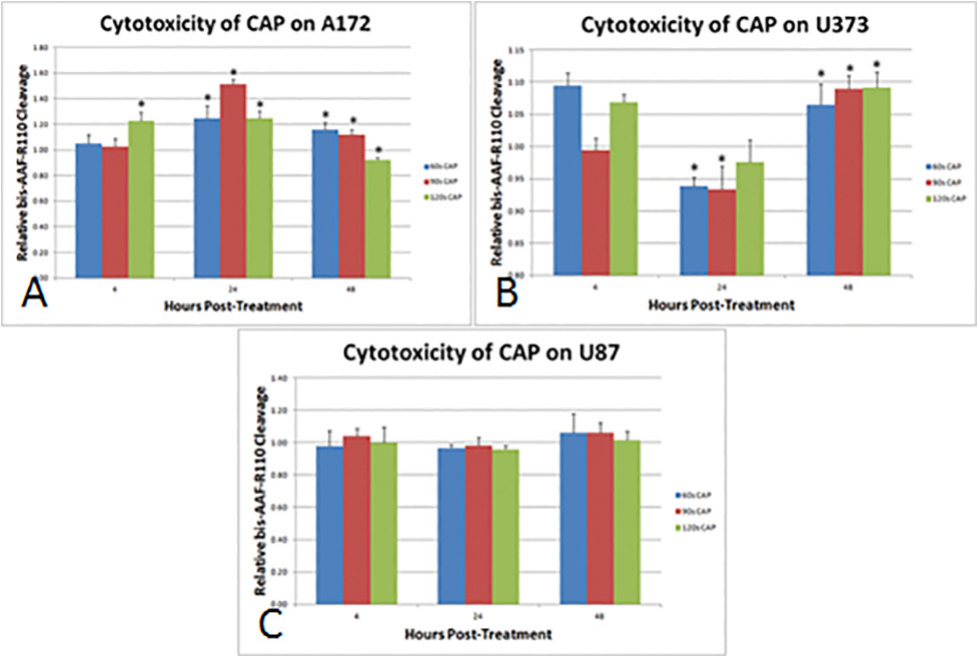
Cytotoxic effects of CAP treatment in three different glioma cell lines. Briefly, cells were treated with CAP for 60, 90 or 120 seconds, incubated with bis-AAF-R110 at 4, 24 and 48 hours post-treatment for 30 minutes for 30 minutes, and emission quantified, as per protocol. bis-AAF-R110 cleavage calculated and plotted relative to untreated control cells. (A) Cytotoxicity was greatest for A172 24 hours post-treatment. (B) Cytotoxicity was greatest for U373 at 3 and 48 hours post-treatment. (C) CAP treatment did not produce a significant amount of cytotoxicity, as measured by bis-AAF-R110 cleavage.* denotes statistical significance compared to control (P <0.05).

Treatment with CAP for 60 seconds resulted in significant cytotoxicity for A172 at 24 and 48 h, and for U373 at 4 and 48 hours (Fig 5). Treatment with CAP for 90 seconds resulted in a statistically significant increase in cytotoxicity for A172 at 24 and 48 hours, U373 at 48 hours. CAP treatment for 120 seconds resulted significant cytotoxicity for A172 at 4 and 24 hours, and for U373 at 4 and 48 hours. No significant cytotoxicity was observed in U87 glioma cells at any time with CAP treatment, indicating again a differential response and the overall resilience of U87 to CAP.

**Fig 6:**
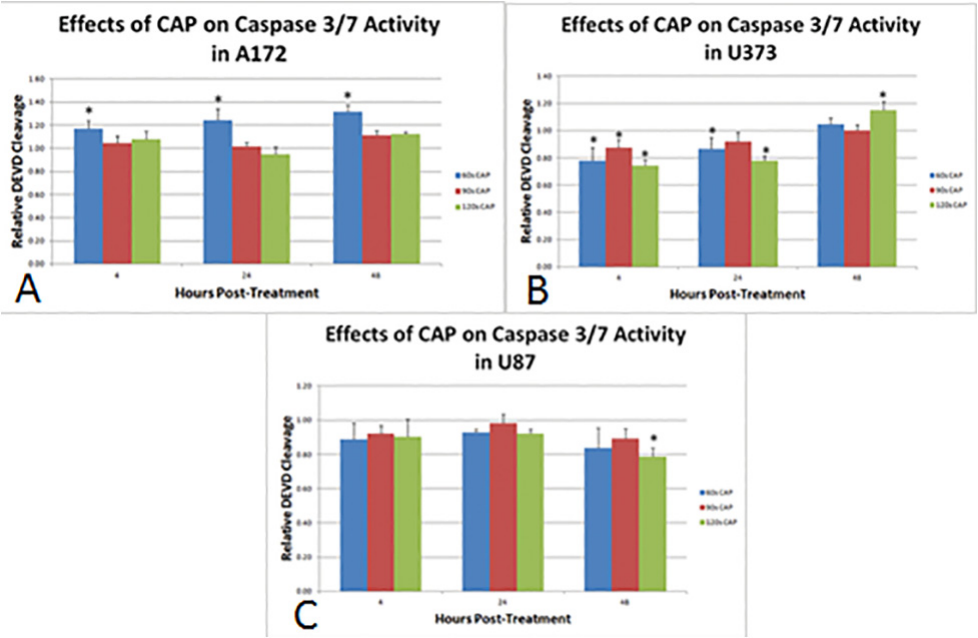
Apoptotic effects of CAP treatment in three different glioma cell lines. Briefly, cells were treated with CAP for 60, 90 or 120 seconds, incubated with DEVD at 4, 24 and 48 hours post-treatment for 30 minutes, and emission quantified, as per protocol. DEVD cleavage calculated and plotted relative to untreated control cells. (A) Treatment with the lowest dosage of CAP, 60s, induced apoptosis in A172. Higher dosages induced cytotoxicity instead of apoptosis. (B) Apoptosis was significantly increased at 48 hours post-CAP at 120 seconds. At 4 and 24 hours post-CAP, a significant decline in caspase 3/7 activity was detected, likely representing a decline in total population due to the cytotoxic effects observed in Fig 3. (C) No significant increase in caspase 3/7 activity was noted with CAP treatment of U87 cells.* denotes statistical significance compared to control (P <0.05).

Apoptosis, as measured by functional caspase 3/7 activity, was also variable across the glioma cell lines (Fig 6). A significant increase in caspase 3/7 activity for A172 was seen at 4, 24, and sustained at 48 hours after 60 seconds treatment with CAP. In U373, caspase 3/7 activity was only increased with 120 seconds treatment at 48 hours post-CAP. A significant decrease in caspase 3/7 activity was noted at 4 and 24 hours post-treatment for 60, 90 and 120 seconds, which complement the findings of cytotoxicity. Caspase 3/7 activity significantly declined compared to control at 24 and 48 hours post-CAP treatment for 120 seconds.

### CAP Treatment Results in a G_2_/M pause Seen in All Glioma Cell Lines

**Fig 7:**
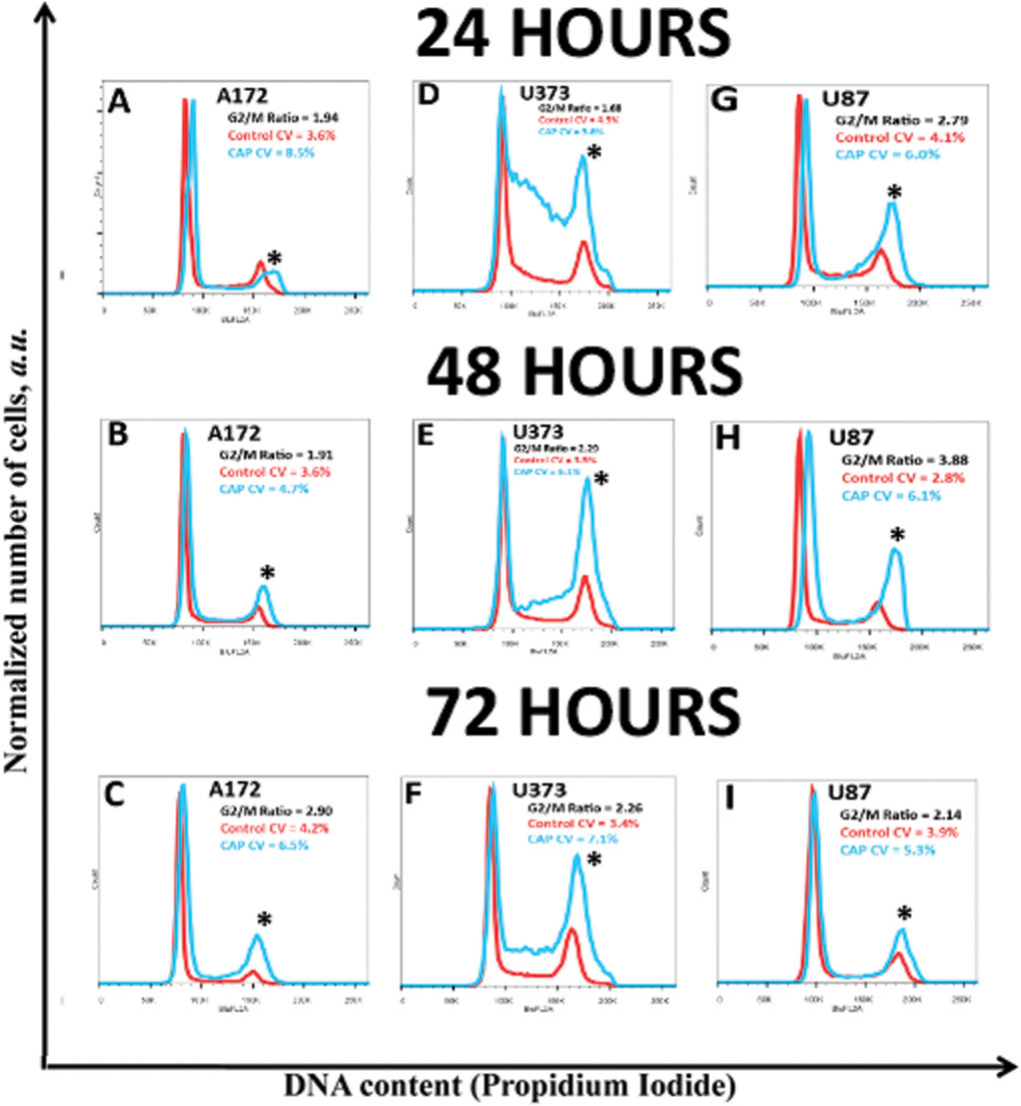
Determination of the cell cycle effect induced by CAP treatment in each glioma cell line. Briefly, each of the glioma cells were treated with 90s CAP, and DNA content was measured at 24, 48, and 72 hours post-CAP treatment. In A172, CAP treatment for 90s resulted in a significant G2/M arrest at (B) 48 and (C) 72 hours, but not at (A) 24 hours. In U373, 90s CAP treatment resulted in a significant increase in the S-phase and G2/M population at (D) 24 hours and sustained at (E) 48 and (F) 72 hours. In U87, CAP treatment for 90s resulted in a significant increase in the G2/M population at (G) 24 and (H) 48 hours that diminished by (I) 72 hours. * denotes statistical significance compared to control (P <0.05).

Consistent with our previous findings [12], CAP treatment manifested a robust increase in the G_2_/M population. Treatment of U87 cells with CAP for 90 and 120 seconds resulted in a greater than two-fold increase in the G_2_/M population at 24 and 48 hours post-treatment (Fig 7). No significant difference was found at 8 and 72 hours post-treatment. Treatment of U373 cells with CAP for 90 seconds increased the G_2_/M and S-phase population at 24 hours, with a sustained greater than two-fold increase in the G_2_/M-phase population at 48 and 72 hours. Treatment of U373 with CAP for 120 seconds resulted in a greater than two-fold increase in the G_2_/M-phase population at 24 and 48 hours post-treatment, with a modest but significant increase in the S-phase population at 24 hours. CAP treatment of A172 cells resulted in a significant increase in the G_2_/M population at 48 and 72 hours.

## Discussion

The unique chemical and physical properties of cold atmospheric plasmas have permitted broad applications in biomedicine, and the recent emergence of the field of plasma healthcare. The most widely established use of plasma is for the sterilization of fragile materials sensitive to thermal damage. Subsequent studies have identified several clinical uses ranging from dental care to various skin diseases such as wound healing, foot fungus, and general hand sterilization without disturbing the skin barrier [26–28]. In contrast to conventional lasers, which are based on thermal-mediated interactions with tissues that result in necrosis and permanent tissue injury, non-thermal plasmas operate at temperatures closer to room temperature to promote focal cell removal without inducing surrounding necrosis. The aim of plasma interaction with tissue is not to denature the tissue but rather to operate under the threshold of thermal damage and to induce chemically specific response modification. In particular, the presence of plasma can promote chemical reactions that would have the desired effects. The plasma jet provides that necessary control for directed treatment within a few millimeters in addition to offering a multifaceted method of cellular injury.

Investigations into the mechanism of injury have pointed to a synergy of several major biologically active components: (1) reactive oxygen species, (2) charge particles, (electric field) (3) UV (primarily UV-C) radiation and (4) ionizing radiation to generate reactive species to cause further damage [1,29]. The substrates that might be affected by the radiation energy are cellular macromolecules (i.e. lipids and proteins) and DNA damage with pyrimidine dimerization [30–33]. Reactive species are inherently unstable, and can directly induce cellular toxicity. These reactive species detected in our cold plasma jet include O_3_, NO, OH radicals, and super oxide radicals. Current research supports the primary effect of the CAP as related to the effect of these free radical species on the tumor cells themselves. Both normal cells and cancer cells are known to display a baseline level of reactive species within the cell membrane. However, this level within cancer cells is considerably higher. Consequently, the threshold at which cells are killed via these free radicals is reached at a much more rapid level.[34–37]. Current research is evaluating the effect of cell media treated via CAP without the presence of plated cells to further elucidate the role of these free radicals. Thus it becomes critical to stringently regulate the distribution of the treatment, which is provided by the plasma jet. However, the true extent of the effects induced by CAP remain to be fully elucidated, as exposure to alternating electrical current can arrest glioblastoma cell proliferation *in vivo* [38] and can also enhance chemotherapy efficacy *in vitro* [39].The effects of cold plasma in eukaryotic cells are diverse and dependent on easily modifiable characteristics such as the treatment duration, power, and the gas composition/pressure used to control chemical reactions [24,40]. Previous studies showed that CAP treatment was highly effective in treating a host of solid tumor malignancies *in vitro* [7–10,35,41–43], with some studies indicating a selectivity for cancerous cells [11,44]. In this pilot study we sought to broadly characterize the efficacy of CAP in three distinct glioblastoma cell lines without damaging normal human astrocytes *in vitro*, with the subsequent goal of transitioning into more complex pre-clinical models.

While prior research displayed a dose dependent effect of CAP on such tumors as colon cancer, our data indicate a dose-dependent effect of CAP on glioma cells, and suggests an optimal treatment duration between 90 to 120 seconds based on the ID_50_ [45]. In general, CAP treatment decreased viability in all three glioma cell lines, with NHA and HUVEC cell lines remaining largely unaffected by 72 hours post-treatment. Consistent with our findings in other malignancies [12], CAP induced a G_2_/M cell cycle pause in all three cell lines. This cell cycle pause is the most likely explanation for the MTT assay results, as the decreased viability may be the result of either decreased cell growth compared to control or an induction of apoptosis to decrease overall cell numbers. An additional explanation could stem from the early or delayed effects of CAP on the mitochondria [37], as dysfunctional mitochondria independent of apoptosis induction could confound the MTT assay results. However, this is unlikely the case especially with the longer treatment duration as the morphologic cell number changes were noted under light microscopy (data not shown).

Among the three glioma cell lines, some variability in sensitivity to CAP treatment is seen based on the MTT assay, with the greatest albeit non-significant resilience seen in the U87 cell line. Examination into the cytotoxic and apoptotic effects also suggests that U87 glioma cells were more resistant to CAP treatment. However, the ID_50_ for U87 remained between 90 and 120 seconds. With regards to the U373 and A172, treatment with CAP resulted in varying but significant degrees of cytotoxicity and caspase 3/7 activation that account for the decreased viability seen with the MTT assay. In specific, U373 exhibited early and delayed cytotoxic effects from CAP with a significant increase in apoptosis observed 48 hours post-treatment. Coupled with the observation that the cell cycle of U373 cells are exquisitely sensitive to CAP treatment, it is likely that the decreased viability in U373 cells are due to early direct effects and also delayed indirect effects induced by CAP. With regards to the A172 cell line, the predominant mechanism of CAP-mediated injury was cytotoxicity, with a significant increase in apoptosis at the lower CAP treatment duration. The results also indicate that varying the duration of CAP treatment can favor either cytotoxicity resulting in necrosis or apoptosis.

Gene expression studies have indicated that glioblastoma are composed of distinct subtypes [45], with differences in pathway dependence. The variance in responses to CAP is likely a manifestation of the distinct entities within glioblastoma. Although it is unknown which subtypes the glioblastoma cell lines are most similar to, it is known that U87 expresses a wild-type p53 whereas U373 and A172 express a mutated form of p53 [46], a key difference that could explain our finding of the resilience of U87 to CAP therapy. Additional analyses involve further elucidation into the key mediators of apoptosis, especially the condition of the mitochondria post-treatment, as previous studies indicate the mechanism of apoptosis is likely mitochondria-mediated [37].

Although more rigorous studies are necessary, CAP may be a promising option to address the limitations of the current treatment algorithm. Glioblastoma is a highly infiltrative tumor that extends beyond the margins of a clinical target volume. Whether it is "micro-infiltration" or en masse infiltration, current therapies can benefit from the addition of a treatment modality specifically directed towards the resection cavity as a great majority of the recurrences are local [47]. Three major obstacles exist in delivering treatments to highly infiltrative solid tumors; (1) treatment penetration, (2) treatment specificity, and (3) tumor cell resistance to treatment. Traditional treatment strategies that utilize vascular delivery are limited by diffusion, and hence are complicated by a stiff extracellular matrix, abnormal vasculature and elevated interstitial fluid pressure which act as significant barriers to drug diffusion and overall poor penetration [48,49]. The specificity of treatment is critical in highly infiltrative tumors where tumor cells are intermixed within normal parenchyma. The third consideration is the tumor's ability to develop resistance to current therapies. The proposed mechanisms of resistance are varied in glioblastoma, and are likely the result of the combination of tumor cell heterogeneity, specifically tumor stem cells [50–52], and it's protective tumor microenvironment [53,54]. The CAP treatment modality does not depend on the vasculature, and thus its penetration is not diffusion dependent. The plasma jet is very focal, which permits a well-defined treatment field. CAP has also been shown to differentially affect tumor cells [11,44], but the specific parameters for glioblastoma remain to be elucidated. In regards to tumor cell resistance, CAP is unique for its multi-modal genotoxic and phototoxic effects, which could provide a distinctive advantage as a more effective treatment. In the special range of 250–300 nm, there are very weak emission lines, which are detected as NO lines. Previously it was shown that the inactivation effect on bacteria by UV-radiation is mostly related to the DNA/RNA damage in UV wavelength of 200–280 nm. Thus, it can be concluded that UV photons are a not major CAP species within the experimental setup [55].

Synergistic effects of UV and ionizing radiation have been previously described [22,23], and the additional treatment of DBD could enhance current chemotherapies by inhibiting MDR drug pumps [39]. While UV photons may not be a key contributor in CAP, the synergistic effect between CAP, radiation therapy and chemotherapeutics remain in be elucidated. The primary limitation in this regard is the standard delay in the surgical application of CAP with subsequent 2–3 weeks for wound healing followed by radiation and chemotherapy. While a clinically applicable CAP device is currently in development, the primary question remains of which patient with glioblastoma will CAP be efficacious. Depth of penetration experiments will be essential to assess the role of CAP in treating the resection cavity following glioblastoma resection. In addition, treatment of unresectable tumors secondary to their relationship to eloquent cortex is of primary interest.

Ultimately, the efficacy of CAP will depend on the ability to optimize the chemical and physical properties of plasmas to maximize therapeutic efficacy with minimal side-effects. This study identifies the optimal treatment duration between 90–120 seconds with our CAP settings, which largely are seen to be effective in decreasing the viability in glioma cells but not in NHA or HUVEC cell lines.

## Conclusion

Treatment of malignant cells with CAP *in vitro* displays promising results in selectively decreasing cell viability in glioma cell lines but not less malignant cell lines (i.e. NHA and HUVEC). The mechanism of tumor inhibition is cell cycle mediated, and depending on the duration of treatment will also induce cytotoxicity resulting in necrosis or apoptosis. These preliminary studies characterize the differential effects of CAP in normal human astrocytes, human endothelial cells, and three distinct glioma cell lines, with minor variability in the mechanism of decreased viability. This study establishes the conditions that will be utilized in the next phase. Future experiments will utilize more complex microenvironments to further elucidate the effects of CAP on glioma progression.
